# Predictors of Long‐Term Response to Ganglion Impar Block With or Without Caudal Epidural Steroid Injection in Chronic Coccydynia: A Retrospective Study

**DOI:** 10.1155/prm/8614330

**Published:** 2026-02-26

**Authors:** Kaan Yavuz, Mesut Bakir, Bedri Ilcan, Ezgi Bercem, Nureddin Teker, Sebnem Rumeli

**Affiliations:** ^1^ Division of Pain Medicine, Department of Anesthesiology and Reanimation, Mersin University Faculty of Medicine, Mersin, 33240, Turkey; ^2^ Department of Anesthesiology and Reanimation, Mersin University Faculty of Medicine, Mersin, 33240, Turkey

## Abstract

**Background:**

Ganglion impar block (GIB) is a commonly used interventional option for refractory chronic coccydynia. Caudal epidural steroid injection (CESI) targets the sacral and coccygeal roots and may theoretically augment the analgesic efficacy of GIB. Evidence comparing GIB alone with a combined GIB and CESI approach, however, remains limited. This study compared long‐term pain outcomes after GIB monotherapy versus GIB combined with CESI.

**Methods:**

In this retrospective single‐center study (Jan 2020–May 2025), we evaluated 56 adults with chronic coccydynia who underwent GIB alone (Group A) or in combination with CESI (Group B). Pain intensity was assessed using the 11‐point numeric rating scale (NRS‐11) at baseline and at 1 h, 1 month, and 6 months after the procedure. Multivariable logistic regression was used to explore predictors of 6‐month response, including early postprocedural pain change and sex.

**Results:**

Significant improvements in pain intensity were observed in both groups and sustained over 6 months. Responder rates at 6 months were comparable between the GIB‐only (41.7%) and GIB with CESI (45.0%) groups (*p* > 0.05). Multivariable analysis revealed that greater early pain relief (baseline to 1 h) significantly predicted 6‐month favorable outcomes (OR: 2.08; 95% CI: 1.26–3.42; *p* = 0.004), whereas male sex was associated with significantly reduced odds of response (OR: 0.11; 95% CI: 0.02–0.72; *p* = 0.022).

**Conclusions:**

In this single‐center retrospective cohort, GIB was associated with sustained pain relief in a substantial proportion of patients with chronic coccydynia, while the addition of CESI did not confer a clear incremental benefit. Importantly, immediate postprocedural pain relief served as a robust predictor of long‐term success, whereas male sex was identified as a negative prognostic factor. Prospective, adequately powered randomized trials are needed to confirm these exploratory findings and to refine patient selection for GIB‐based interventions.

**Trial Registration:** ClinicalTrials.gov Identifier: NCT07200765

## 1. Introduction

Coccydynia is a chronic pain syndrome localized to the coccygeal region. The pain typically worsens with sitting and is frequently most intense when rising from a seated position [1]. This condition is more common in women and middle‐aged individuals [2]. The pain can make everyday tasks difficult and may significantly affect a person’s overall well‐being [3].

The etiology of coccydynia often remains unclear, although the condition is frequently associated with trauma, vaginal delivery, obesity, and repetitive mechanical stress [4]. Current guidelines recommend conservative approaches as first‐line treatment. These primarily include nonsteroidal anti‐inflammatory drugs (NSAIDs), seating cushions, and physical therapy [5]. For patients who do not respond to these initial measures, interventional procedures are then indicated [6].

The ganglion impar block (GIB) was first described for palliative management of malignant perineal pain and is now widely used in benign coccydynia [7]. By targeting sympathetic fibers in the sacrococcygeal region, this intervention represents a reasonable therapeutic approach, as it acts directly on anatomical structures responsible for transmitting coccygeal pain [8]. Although short‐term analgesic benefit is well documented, evidence on long‐term efficacy is scarce and inconsistent [9].

Caudal epidural steroid injection (CESI) is an alternative interventional modality that may offer a theoretical advantage in the treatment of coccydynia because of its potential to spread to the sacral and coccygeal nerve roots [10]. As it targets different anatomical sites than the GIB, it has been proposed that their combined application could enhance clinical effectiveness [11]. However, the current literature on this combined approach is sparse, and it remains unclear whether CESI provides any additional benefit over GIB alone [12].

The primary aim of this study was to determine whether adding CESI to GIB improves 6‐month pain outcomes compared to GIB alone in refractory chronic coccydynia; a secondary aim was to identify clinical predictors of long‐term response.

## 2. Methods

### 2.1. Study Design and Participants

This retrospective study was approved by the Mersin University Clinical Research Ethics Committee (Decision No: 2025/605, Date: May 28, 2025). The study cohort included patients who presented to the outpatient pain clinic with chronic coccydynia between January 1, 2020, and May 20, 2025, and who underwent a GIB.

**FIGURE 1:**
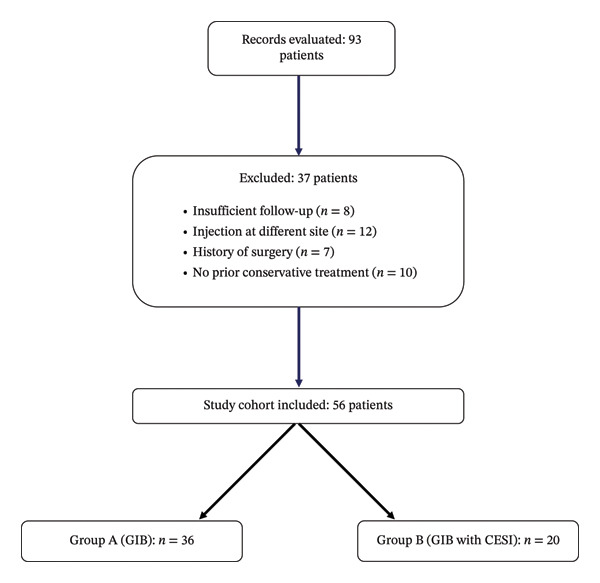
Flowchart.

After the initial evaluation of 93 patients, 37 were excluded: 7 due to a history of lumbar or coccygeal surgery, 8 because of insufficient follow‐up data, 12 because an additional injection was administered at another site during the same session, and 10 due to lack of prior conservative treatment (Figure 1). Consequently, 56 patients were included in the final analysis.

Inclusion criteria were as follows: a diagnosis of chronic coccydynia lasting ≥ 3 months, failure of prior conservative management, age ≥ 18 years, and provision of written informed consent for the procedure. Exclusion criteria were as follows: active local or systemic infection, history of lumbar or coccygeal surgery, known allergy to local anesthetics or contrast media, and coagulopathy or bleeding diathesis.

#### 2.1.1. Interventions

The decision to perform GIB alone or in combination with CESI was based on physician preference and routine clinical practice during the study period, rather than predefined selection criteria.

#### 2.1.2. Group A (GIB Alone)

**FIGURE 2:**
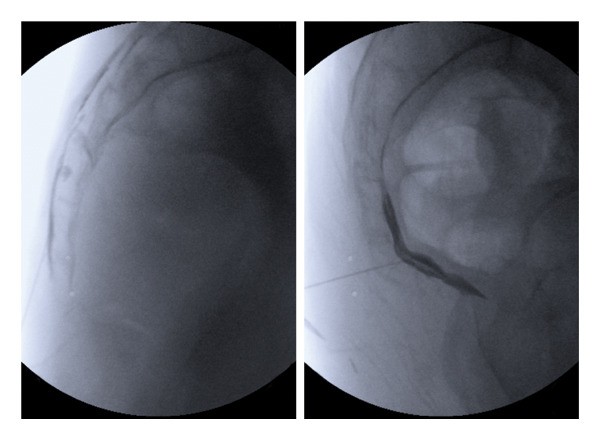
Fluoroscopic images demonstrating the GIB and GIB with CESI. (a) Lateral view of the CESI, illustrating the needle positioned in the caudal epidural space with appropriate epidural spread of 1 mL iohexol contrast. (b) Lateral view showing the transsacrococcygeal approach for GIB, with the needle tip positioned anterior to the sacrococcygeal ligament and correct placement confirmed by injection of 1 mL iohexol contrast into the ganglion impar region.

The GIB procedure was performed under fluoroscopic guidance with the patient in the lateral position, using a transsacrococcygeal approach. Following the loss‐of‐resistance technique, the needle was advanced toward the sacrococcygeal junction, with its tip positioned anterior to the sacrococcygeal ligament. Correct placement was confirmed using 1 mL of iohexol contrast agent (350 mg/mL; Omnipaque 350, GE Healthcare, Ireland). Subsequently, a 5 mL solution containing 40 mg (1 mL) methylprednisolone acetate, 2 mL of 0.9% NaCl, and 2 mL of 0.5% bupivacaine was injected (Figure 2).

#### 2.1.3. Group B (GIB with CESI)

In Group B, patients first underwent a CESI under fluoroscopic guidance, utilizing both lateral and anteroposterior (AP) views. After subcutaneous infiltration with 1 mL of 2% lidocaine for local anesthesia, a 22‐gauge spinal needle was introduced into the caudal epidural space. Its correct positioning was verified with 1 mL of iohexol contrast agent. A 5 mL solution of 40 mg methylprednisolone acetate diluted in 4 mL of 0.9% NaCl was then administered. Immediately following the CESI procedure, a standard GIB was performed in the same session, as described for Group A (Figure 2).

### 2.2. Data Collection

Demographic and clinical data were extracted from medical records, including age, sex, comorbidities, history of coccygeal trauma, and duration of pain. Pain intensity was assessed using the 11‐point numeric rating scale (NRS‐11), where 0 indicates “no pain” and 10 indicates “the worst pain imaginable.” NRS‐11 scores were recorded at baseline (preprocedure), 1 h postprocedure, and at 1 and 6 months.

In line with the Initiative on Methods, Measurement, and Pain Assessment in Clinical Trials (IMMPACT) recommendations, a clinically important improvement was defined as either a ≥ 50% reduction or an absolute decrease of ≥ 4 points in NRS‐11 at the 6‐month follow‐up. Patients meeting this criterion were classified as responders, and the remainder as nonresponders [13].

### 2.3. Statistical Analysis

Statistical analyses were performed using SPSS Version 20.0 (IBM Corp., Armonk, NY). The normality of continuous variables was evaluated using the Kolmogorov–Smirnov and Shapiro–Wilk tests. Data are presented as mean ± standard deviation for normally distributed variables, median (minimum–maximum) for non‐normally distributed variables, and number (percentage) for categorical variables.

Intergroup comparisons were conducted using the Student’s *t*‐test for normally distributed continuous variables, the Mann–Whitney *U* test for non‐normally distributed continuous variables, and the Pearson chi‐square or Fisher’s exact test for categorical variables, as appropriate.

To identify independent predictors of treatment response, variables with a *p* value of < 0.05 in the univariate analyses were included in a binary logistic regression model using the enter method. The results of the regression analysis are reported as odds ratios with 95% confidence intervals. A two‐tailed *p* value of < 0.05 was considered statistically significant.

## 3. Results

The analysis included 56 patients (mean age: 51.4 ± 16.8 years; 76.8% female). Of these, 36 were in the GIB group (Group A), and 20 were in the GIB with CESI group (Group B). The groups demonstrated comparable baseline characteristics, including age, sex, comorbidities, pain duration, and trauma history (Table 1). No serious procedure‐related adverse events were observed in either group.

**TABLE 1:** Baseline demographics and clinical characteristics of study participants (*n* = 56).

	All patients (*n* = 56)	GIB with CESI (*n* = 20)	GIB alone (*n* = 36)	*p*
Age (years), mean ± SD (min–max)	51.39 ± 16.84 (21–85)	49.25 ± 16.05 49 (21–78)	52.58 ± 17.37 54.50 (21–85)	0.483
Sex, *n* (%)				0.370
Female	43 (76.8)	14 (70)	29 (80.6)	
Male	13 (23.2)	6 (30)	7 (19.4)	
Coexisting conditions, *n* (%)				0.447
Hypertension	11 (19.6)	5 (25.0)	6 (16.6)	
Diabetes	5 (8.9)	2 (10.0)	3 (8.3)	
Trauma history, *n* (%)	30 (53.6)	11 (55.0)	19 (52.8)	0.873
Pain duration (months)	9.16 ± 4.80	9.45 ± 5.67	9.00 ± 4.32	0.925
NRS scores, mean ± SD (min–max)				
Preoperative	7.21 ± 1.09 7.00 (3–9)	7.30 ± 0.73 7.00 (6–8)	7.17 ± 1.25 7.00 (3–9)	0.942
Postoperative	2.64 ± 1.49 2.00 (1–8)	3.10 ± 1.94 3.00 (1–8)	2.39 ± 1.12 2.00 (1–6)	0.226
1‐Month	3.98 ± 1.87 4.00 (1–8)	3.90 ± 2.17 4.00 (1–8)	4.02 ± 1.71 4.00 (1–8)	0.809
6‐Month	4.55 ± 2.23 4.00 (0–8)	4.60 ± 2.47 4.00 (0–8)	4.53 ± 2.11 4.50 (0–8)	0.913

Abbreviations: CESI, caudal epidural steroid injection; GIB, ganglion impar block; NRS, numeric rating scale.

**FIGURE 3:**
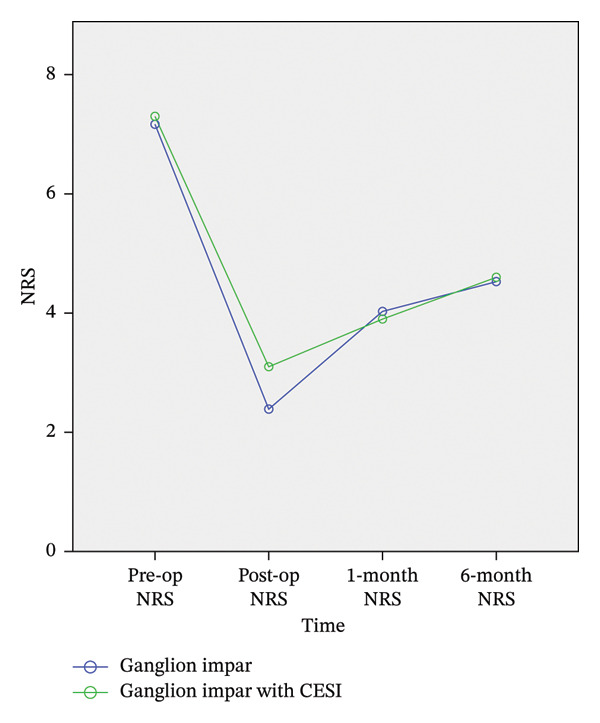
The changes in the NRS of the two groups.

The baseline NRS scores were similar between Group A (7.17 ± 1.25) and Group B (7.30 ± 0.73; *p* = 0.942). Pain scores decreased significantly in both groups following the intervention and remained improved throughout the 6‐month follow‐up period. Accordingly, the intergroup analysis revealed no statistically significant differences in NRS scores at 1 h (*p* = 0.226), 1 month (*p* = 0.809), or 6 months (*p* = 0.909) postprocedure (Table 1) (Figure 3).

According to IMMPACT criteria, 15 patients (41.6%) in Group A and 9 (45.0%) in Group B were classified as treatment responders. Among these responders, the postprocedural NRS scores were comparable, with no statistically significant difference between the groups (1 h, 1 month, and 6 months) (Table 2).

**TABLE 2:** Comparison of baseline clinical and demographic characteristics between the responder and nonresponder groups.

Variables	Responders	Nonresponders	*p*
Age	49.54 ± 17.79	52.78 ± 16.24	0.488
Sex, *n* (%)			0.028
Female	22 (91.7)	21 (65.6)	
Male	2 (8.3)	11 (34.4)	
Pain duration (months)	8.66 ± 5.45	9.53 ± 4.30	0.200
Trauma history *n* (%)	12 (50.0)	18 (56.3)	0.043
Coexisting conditions, *n* (%)			0.625
Hypertension	4 (16.7)	7 (21.9)	
Diabetes	2 (8.4)	3 (9.4)	
Preoperative NRS	7.63 ± 0.64	6.91 ± 1.25	0.015
1‐Month NRS	2.29 ± 0.99	5.25 ± 1.27	0.001
6‐Month NRS	2.54 ± 1.28	6.06 ± 1.45	0.001
Injection types (%)			0.809^∗^
GIB	15 (41.6)	21 (58.4)	
GIB with CESI	9 (45.0)	11 (55.0)	

Abbreviations: CESI, caudal epidural steroid injection; GIB, ganglion impar block; NRS, numerical rating scale.

^∗^No significant differences in NRS scores were observed among responders at baseline, immediately postprocedure, 1 month, or 6 months (*p* = 0.953, 0.726, 0.129, and 0.550, respectively).

In separate bivariate logistic regression analyses, age, pain duration, and history of trauma were not significantly associated with treatment response at 6 months. The odds ratio for age was 0.988 (95% CI: 0.957–1.020; *p* = 0.474), for pain duration 0.962 (95% CI: 0.857–1.078; *p* = 0.504), and for absence versus presence of a history of trauma 0.778 (95% CI: 0.269–2.250; *p* = 0.643). As none of these variables reached statistical significance (all *p* > 0.05), they were not included in the multivariable model.

The multivariable logistic regression analysis examining predictors of clinical response at 6 months was significant (Omnibus *χ* ^2^ = 18.69, *p* = 0.001; Hosmer–Lemeshow goodness‐of‐fit test *p* = 0.940). The model’s overall classification accuracy was 73.2%. Among the independent variables, the change in NRS (from preoperative to 1 h) was identified as a significant predictor; each one‐point decrease was associated with a 2.07‐fold increase in the likelihood of clinical response (OR: 2.075; 95% CI: 1.261–3.416; *p* = 0.004). In contrast, male sex significantly reduced the likelihood of treatment response (OR: 0.109; 95% CI: 0.017–0.723; *p* = 0.022) (Table 3).

**TABLE 3:** Multivariable logistic regression analysis for predictive factors associated with successful response after ganglion impar with or without CESI injection.

Variables	Univariate		Multivariate	
	OR (%95 CI)	*p*	OR (%95 CI)	*p*
ΔNRS	1.875 (1.211–2.903)	0.005	2.075 (1.261–3.416)	0.004
Sex female (ref.) male	0.174 (0.034–0.878)	0.034	0.109 (0.017–0.723)	0.022

*Note:* ΔNRS, the change in NRS (from preoperative to 1 h). Cox and Snell R‐square: 0.284, Nagelkerke R‐square: 0.381, and accuracy: %73.2.

## 4. Discussion

This retrospective study investigated the contribution of adding CESI to GIB on clinical efficacy in patients with chronic coccydynia refractory to conservative treatment. Our findings demonstrated that both GIB and GIB with CESI applications provided a significant reduction in pain intensity; however, no statistically significant difference was found in short‐ and medium‐term NRS scores between the groups. Furthermore, the difference in NRS scores and sex was identified as a significant predictor influencing the treatment response.

The effectiveness of GIB in the treatment of coccydynia has been supported by numerous studies in recent years. In the meta‐analysis conducted by Jevotovsky et al., data from 18 studies including more than 600 patients were evaluated, and GIB was shown to provide significant pain reduction in the short (≤ 3 months), medium (3–6 months), and long (> 6 months) terms. However, due to study heterogeneity and low methodological quality, the overall level of evidence was reported as “very low” [14]. Studies by Nasiri et al. and Olgun et al. reported statistically significant reductions in pain scores and high patient satisfaction following GIB [15, 16]. Nevertheless, considering the effectiveness of GIB, it does not appear to confer absolute superiority when compared to alternative methods. Indeed, the randomized clinical trial by Gene and Yıldız demonstrated that ultrasound‐guided coccygeal nerve block achieved pain and functional improvement comparable to that of GIB [17].

Only a small number of studies have evaluated CESI for coccydynia. In a randomized trial comparing GIB with CESI, Sencan et al. reported that both interventions reduced pain on the NRS; GIB showed greater early improvement at 3 weeks, although the difference was not clinically meaningful by 3 months [3]. In a retrospective comparison of GIB versus combined GIB with CESI, Samet Sancar et al. found no additional benefit of the combination, with similar pain control after adjustment for baseline covariates [18]. Aligning with previous research, our results confirm that adding CESI failed to yield significant additional clinical benefit.

CESI offers a theoretical advantage due to its potential for medication distribution to the sacral nerve roots, suggesting it may influence different anatomical structures than GIB. Nevertheless, robust clinical evidence confirming a clear synergistic benefit from this mechanism is lacking. In a recent investigation, Gazioğlu and Rumeli observed that combining ganglion impar radiofrequency with CESI showed no superiority over ganglion impar pulsed radiofrequency alone, with comparable pain control outcomes at the 6‐month assessment. Curiously, patients receiving combination therapy reported subjectively higher satisfaction levels [11]. While some reports suggest that CESI could provide benefits for specific patient subgroups, particularly in cases of postpartum coccydynia, its general efficacy across broader patient populations still requires further validation [19].

Multivariable analysis identified two independent predictors of treatment response: the magnitude of early pain reduction and patient’s sex. Specifically, for each one‐point decrease in the NRS score from baseline to the first hour postprocedure, the odds of achieving a clinical response at 6 months more than doubled (OR: 2.07). This finding is consistent with a report by Şencan et al., who observed a similar relationship between early pain reduction and long‐term outcome following transforaminal epidural steroid injections [20]. Conversely, male sex demonstrated a significant association with reduced treatment success. The influence of sex on response to GIB has received limited attention in the existing literature, which makes our observation an important and novel contribution. Potential underlying reasons may include anatomical variations in the pelvic floor, the influence of hormonal factors, or sex‐specific differences in pain perception; these hypotheses merit further dedicated investigation.

There is growing recognition that functional outcomes and quality of life represent critical endpoints in coccydynia management. Supporting this view, Sencan et al. demonstrated that patients undergoing GIB showed significantly better early quality‐of‐life outcomes and greater relief from neuropathic pain components [3]. A parallel trend appears in studies evaluating CESI, in which patients frequently report higher satisfaction despite no superior improvements in pain scores [11]. This consistent disconnect between objective pain measures and subjective patient experience suggests that a comprehensive evaluation of therapeutic success should integrate key functional parameters, such as sitting tolerance, daily activity performance, and overall quality of life, alongside conventional pain assessment.

Coccydynia management includes the traditional GIB alongside newer techniques such as pulsed radiofrequency and platelet‐rich plasma (PRP) injections. Preliminary research by Pilco et al. indicates that PRP might provide better functional improvement than PRF in treatment‐resistant cases [21]. Still, properly conducted randomized trials comparing these interventions directly are needed to clarify their relative benefits. Future investigations along these lines would substantially enhance clinical guidance, helping practitioners select optimal therapies for specific patient presentations.

This study has several limitations. The retrospective design entails risks of documentation errors and selection bias. The limited sample size, particularly in the combination therapy group, restricted the statistical power of the multivariable regression analysis and raises the possibility of overfitting. Therefore, the identified predictors should be interpreted as exploratory findings. Pain severity was assessed solely with a subjective scale (NRS), and the absence of functional measures reduces the interpretability of the findings. Potentially relevant variables, such as anatomical variation (coccygeal morphology and mobility), were not analyzed in this study.

In conclusion, our findings confirm the efficacy of GIB for chronic coccydynia while demonstrating that the addition of CESI provides no significant clinical advantage. Future investigations should prioritize elucidating the intriguing relationships between early pain reduction, patient sex, and treatment response. There is a need for large‐sample, randomized, controlled trials with long‐term follow‐up, and these studies should also include functional and quality‐of‐life measures among the evaluation parameters.

## Funding

The authors received no financial support for the research, authorship, and/or publication of this article.

## Conflicts of Interest

The authors declare no conflicts of interest.

## Data Availability

Data are available from the corresponding author on reasonable request.
